# Intradural Extramedullary Capillary Hemangioma in the Upper Thoracic Spine: A Review of the Literature

**DOI:** 10.1155/2014/604131

**Published:** 2014-06-18

**Authors:** Yoichiro Takata, Toshinori Sakai, Kosaku Higashino, Yuichiro Goda, Fumitake Tezuka, Koichi Sairyo

**Affiliations:** Department of Orthopedic Surgery, The University of Tokushima, 3-18-15 Kuramoto-cho, Tokushima 770-8503, Japan

## Abstract

Capillary hemangiomas are benign tumors found in the skin and soft tissues in younger people. They occur in the central nervous system only rarely, and intradural occurrence is extremely rare. We report here a 60-year-old man presenting with thoracic girdle pain and progressive gait disturbance. Magnetic resonance images of the thoracic spine showed a 12 × 8 × 20 mm, well-defined intradural mass at the T2 level, compressing the spinal cord laterally. Relative to the spinal cord, the mass was hypo- to isointense on T1-weighted images and relatively hyperintense on T2-weighted images, with strong enhancement on contrast-enhanced T1-weighted images. The patient underwent T1-2 hemilaminectomy with resection of the intradural extramedullary tumor, which showed characteristics of a capillary hemangioma on histologic examination. The patient's symptoms improved following the surgery and no clinical or radiological evidence of recurrence was noted at the 2-year follow-up. We present this case with a review of the literature, highlighting features for differential diagnosis.

## 1. Introduction

Capillary hemangiomas are benign vascular malformations, most often found in the skin or soft tissue throughout the body in younger patients. They are histologically characterized by nodules of capillary-sized vessels lined by flattened endothelium [1]. Capillary hemangiomas in the central nervous system are rare, and intradural occurrence is extremely rare. To the best of our knowledge, 35 cases of pure intradural extramedullary capillary hemangioma have been reported to date [2–20].

On magnetic resonance imaging (MRI), these lesions appear isointense and hyperintense relative to the spinal cord on T1-weighted and T2-weighted images, respectively, and exhibit a strong homogeneous enhancement on contrast-enhanced T1-weighted images. Common intradural spinal tumors such as schwannoma and meningioma have similar MRI features [21].

In this report, a case of intradural extramedullary capillary hemangioma in the upper thoracic spine is described and the literature is reviewed.

## 2. Case Presentation

A 60-year-old man presented with a 2-month history of thoracic girdle pain, followed by 1 week of progressive gait disturbance. Physical examination revealed no abnormalities. On neurologic examination, there were no muscle weakness and no sensory disturbance, except for loss of vibration sensation below the knee. The patient was continent of urine.

**Figure 1:**
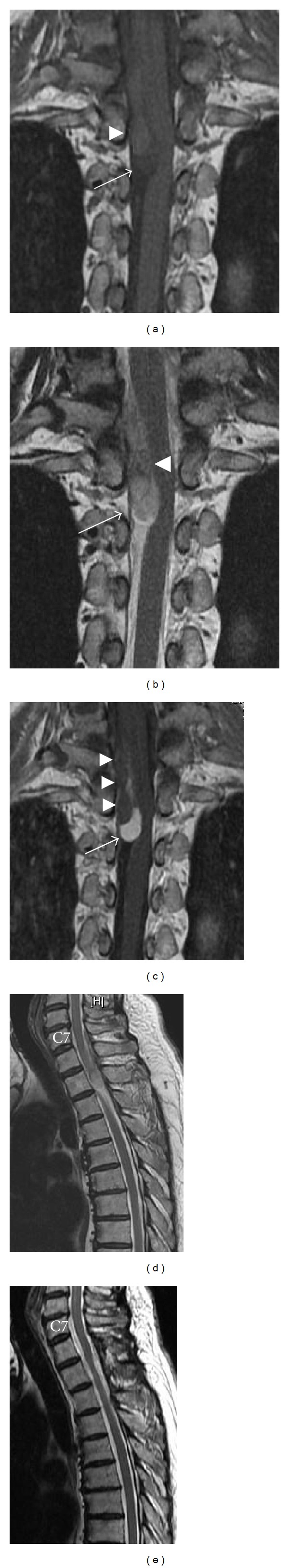
Preoperative magnetic resonance images. (a) Coronal T1-weighted image shows a round well-circumscribed lesion with hypointensity (arrow) and isointensity (arrowhead) relative to the spinal cord at the T2 level. (b) Coronal T2-weighted image shows a relatively hyperintense lesion (arrow) compressing the spinal cord laterally. A vascular-like structure is apparent cranial to the mass, indicating enlarged vessels (arrowhead). (c) Contrast-enhanced T1-weighted coronal image shows strong enhancement (arrow) in the caudal part of the mass and a moniliform structure without enhancement (arrowhead) in the cranial part of the mass. (d) Sagittal T2-weighted image shows a well-circumscribed lesion with heterogeneous hyperintensity. (e) Postoperative sagittal T2-weighted image shows complete resection of the tumor.

MRI of the thoracic spine showed a 12 × 8 × 20 mm, well-defined intradural mass at the T2 level, compressing the spinal cord laterally. Relative to the spinal cord, the mass was hypo- to isointense on T1-weighted images and relatively hyperintense on T2-weighted images. Also shown on T2-weighted images was a structure in the cranial aspect of the mass that was consistent with enlarged vessels. The caudal part of the mass showed strong enhancement on contrast-enhanced T1-weighted images and the cranial part of the mass showed a moniliform structure without enhancement (Figure 1). The preoperative diagnosis was a neurogenic tumor or vascular malformation.

The patient underwent T1-2 hemilaminectomy. On opening the dura, a well-circumscribed, dark reddish mass was seen beside the spinal cord. The mass was adherent to the arachnoid and nerve root, especially on the left side. Under an operating microscope, the mass was dissected out from the spinal cord and nerve root. The cranial part of the mass consisted of dilated vessels and was resected completely with cauterization shrinkage without significant bleeding.

**Figure 2:**
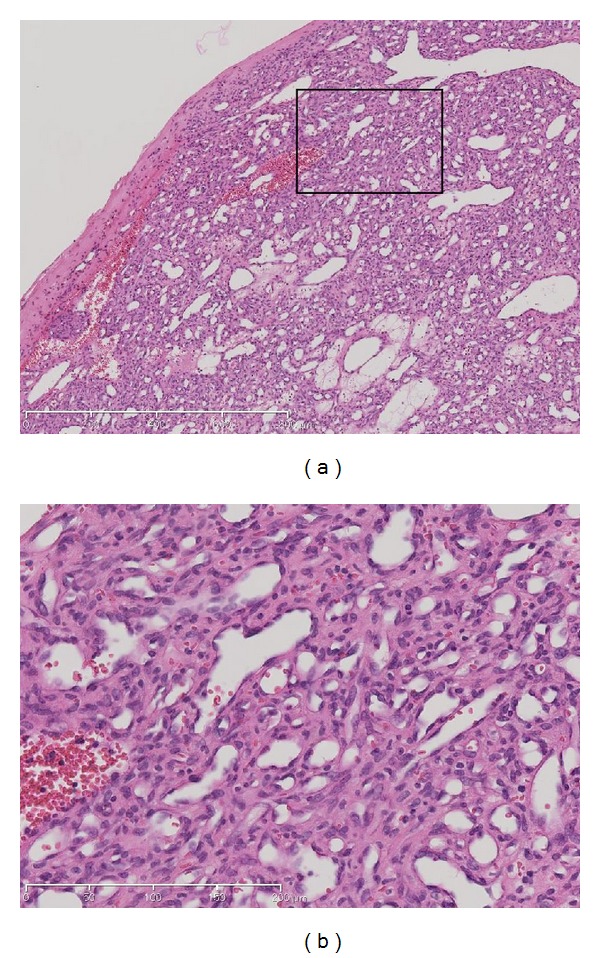
Photomicrograph of the lesion reveals a lobule composed of small capillary vessels lined by a layer of endothelial cells. (a) A lobular architecture with numerous capillary-sized vessels lined by a single layer of endothelial cells and dissemination of dilated vessels. Hematoxylin and eosin stain, with original magnification ×5. (b) Various sizes of capillary vessels lined by flattened endothelium. Hematoxylin and eosin stain, with original magnification ×20.

The surgical specimen was fixed in 10% buffered formalin, routinely processed, and embedded in paraffin. Sections were prepared and stained with hematoxylin and eosin. Histological examination revealed that the mass had a lobular architecture with numerous capillary-sized vessels lined by a single layer of endothelial cells and dissemination of dilated vessels (Figure 2). Immunohistochemical staining was performed using monoclonal antibodies against inhibin, CD56, and S100 to rule out hemangioblastoma, schwannoma, and neurofibroma. Immunohistochemical staining for each antibody was negative. All of these features were consistent with a capillary hemangioma.

Although the patient experienced sensory disturbance of the right T3 dermatome postoperatively, his thoracic girdle pain disappeared immediately after surgery and his gait disturbance improved gradually. Although the right T3 sensory deficit persisted, there was no clinical or radiological evidence of recurrence at the 2-year follow-up (Figure 1).

## 3. Discussion

Spinal cord tumors account for about 15% of all central nervous system neoplasms. Vascular lesions comprise about 6-7% of all spinal intradural tumors [1] and commonly include cavernous and capillary hemangiomas. In the intradural extramedullary space, hemangiomas may arise from the blood vessels of the nerve roots in the cauda equina, the inner surface of the dura, or the pial surface of the spinal cord [2–7, 21, 22]. They can be differentiated histologically by vessel size [21]. Cavernous hemangiomas are comprised of irregular, dilated sinusoidal vascular channels lined by a monolayer of benign endothelium, while capillary hemangiomas are encapsulated lesions characterized by nodules of capillary-sized vessels lined by flattened endothelium [1, 23].

Capillary hemangiomas usually occur distantly, at the conus medullaris or attached to nerve roots of the cauda equina [1]. Of the 35 cases of pure intradural extramedullary capillary hemangioma reported (Table 1) [2–20], intradural extramedullary capillary hemangiomas tended to present in the fourth or fifth decade of life (mean age: 49 years). Previous review articles reported a male-to-female ratio of 1 : 1 [1]. However, in our review of the literature, this ratio was 3 : 1, with male predominance (Table 1). The lesions were in the thoracic spine in 15 of the 35 cases and in the lumbar or conus medullaris region in the remaining cases. The thoracic lesions were located between T4 and T11 vertebrae [5–7, 9–13, 16, 18, 20]. Our case, which occurred at the T2 level, appears to be the most cranial case. The preoperative symptoms of these lesions are variable and can include low back pain, radiating leg pain, motor weakness, gait disturbance, and urinary incontinence. Almost all patients presented with back or low back pain. Roncaroli et al. reported a female patient with episodic leg pain that was temporally related to menses [5]. In the previously reported cases, over 90% experienced improvement postoperatively. However, Nowak et al. reported a case at the level of T12/L1 that was complicated by postoperative muscle weakness persisting for 14 months [8]. In the operative findings in their case, microsurgical dissection of the nerve fibers densely adherent to the tumor was not possible without scarifying them.

The MRI findings in the present case are consistent with those of previous reports; that is, the lesion appeared isointense relative to the spinal cord on T1-weighted images and iso- or hyperintense on T2-weighted imaging with strong homogeneous gadolinium enhancement. However, shown on T2-weighted images was a structure in the cranial aspect of the mass that was consistent with enlarged vessels. We were not able to rule out the vascular malformation by only MRI findings. However, we did not perform preoperative angiography. In the literatures, most intradural capillary hemangiomas were resected successfully without significant intraoperative bleeding, although some cases showed high bleeding tendency [6, 10, 15, 17]. In our case, fortunately, tumor was resected without significant bleeding. To avoid intraoperative bleeding, preoperative angiography and/or embolization should be considered [16].

The most common intradural extramedullary tumors are schwannoma and meningioma, both of which show marked enhancement on contrast-enhanced T1-weighted images. Schwannomas are usually hypointense on T1-weighted images and hyperintense on T2-weighted images, with no evident infrequent cystic or necrotic changes. Without these findings, it is difficult to differentiate schwannoma from hemangioma. However, in the present case, the vascular-like structure, which was cranial to the mass, suggested the presence of vascular lesions. Meningioma has an isointense appearance on T1-weighted images and an iso- or hyperintense appearance on T2-weighted images. The dural tail sign is not useful in distinguishing meningioma, since a capillary hemangioma may arise from the inner surface of the dura mater creating a dural tail sign [9]. It is impossible to differentiate intradural extramedullary capillary hemangioma from other common intradural tumors by MRI. In previously reported cases, meningioma or schwannoma was the preoperative diagnosis, based on the findings of MRI (Table 1) [2, 3, 9, 10, 13, 15, 17].

## 4. Conclusion

Intradural extramedullary capillary hemangioma is rare and may be clinically or radiologically indistinguishable from other lesions, but they should be considered when making a differential diagnosis of intradural extramedullary neoplasms.

## Figures and Tables

**Table 1:** Review of the literature on intradural extramedullary capillary hemangioma.

Author/year	Patient age/sex	Location (number of cases)	Symptom	Preoperative diagnosis	Prognosis	F/U period	Recurrence
Babu et al. 2013 [20]	53.5 (mean): 4 cases	Thoracic (2) Lumbar (2)	Pain, weakness, sensory abnormalities, UI	Not mentioned	Worse in 20%	Not shown	No recurrence

Sonawane et al. 2012 [19]	35/M	Conus	LBP, weakness	Not mentioned	Neurological improvement	1.5 years	Not shown

Kaneko et al. 2012 [18]	48/M	T10-11	LBP, GD	Not mentioned	Rapid sensorimotor improvement	9 years	Regrowth 6 months after operation

Funayama et al. 2010 [17]	34/M	L4	LBP, leg pain	Cauda equina neurinoma	No symptoms at 1 Y F/U	1 year	No recurrence

Chung et al. 2010 [16]	47/M	T6-7	LBP, leg pain	Not mentioned	Sensory impairment gradually improved	Not shown	Not shown

Miri et al. 2009 [15]	20/M	L3	LBP, leg pain, weakness, UI	Cauda equina neurinoma	Weakness and urogenital problems improved	1 year	No recurrence

Kim et al. 2006 [14]	59/M	L1-2	LBP, leg pain	Not mentioned	Pain was improved	Not shown	Not shown

Yu et al. 2006 [13]	48/M	T6-7	BP, leg pain	Neurogenic tumor or meningioma	BP improved weakness recovered	2 months	Not shown

Alakandy et al. 2006 [12]	60/M	T9	BP, weakness, leg pain	Not mentioned	Neurological improvement	Not shown	Not shown

Abe et al. 2004 [11]	59/M	T11	Paraparesis	Not mentioned	Recovery from symptoms	1 year	No recurrence

Abdullah et al. 2004 [10]	32/F	T10	LBP, weakness, GD	Neurinoma, neurofibroma, meningioma, hemangioblastoma, paraganglioma	Neurological improvement	Not shown	Not shown

Choi et al. 2001 [9]	28/M 52/M 51/M	L1 T5-6 T4-5	BP, weakness claudication, weakness claudication, leg pain	Neurinoma or meningioma Meningioma Meningioma	Not mentioned	Not shown	Not shown

Nowak et al. 2000 [8]	63/F	Conus	LBP, numbness	Not mentioned	Residural paresis left tibialis anterior on 14-month F/U	14 months	Not shown

Roncaroli et al. 2000 [7]	42/F 50/M 53/M 64/M	T11 T11 Conus T10	Weakness LBP, weakness BP Leg pain, weakness	Preoperative diagnosis of hemangioma was not made	Recovery little improvement Leg weakness Recovery	11 years 2 years 1.5 years 9 years	Not shown

Shin et al. 2000 [6]	66/F	T8-9	LBP, weakness	Not mentioned	Weakness and LBP improved	6 months	Not shown

Roncaroli et al. 1999 [5]	40–62/6 M & 3 F	T5 (1) Lumbar (8)	Leg pain, weakness	Not mentioned	Relieving leg painimproved weakness	Not shown	No recurrence

Zander et al. 1998 [4]	51/F	L4-5	LBP, leg pain	Not mentioned	Postoperative course was uneventful	Not shown	Not shown

Mastronardi et al. 1997 [3]	41/M	L5	LBP, leg pain	Cauda equina neurinoma	Pain was improved	Not shown	Not shown

Hanakita et al. 1991 [2]	58/M	L1-2	LBP, leg pain	Cauda equina neurinoma	Not mentioned	Not shown	Not shown

BP: back pain; LBP: low back pain; GD: gait disturbance; UI: urinary incontinence; F/U: follow-up.
